# East Asian multi-centre study on clinical long-term outcomes of postoperative proton beam therapy for localised paediatric intracranial ependymoma

**DOI:** 10.1016/j.ctro.2026.101270

**Published:** 2026-08-22

**Authors:** Akifumi Kajihara, Yusuke Demizu, Takashi Saito, Yusuke Azami, Shosei Shimizu, Sae Matsumoto, Yuichi Hiroshima, Hiromitsu Iwata, Norihiro Aibe, Hideyuki Sakurai, Masao Murakami, Gao Zeng, Hiroyasu Tamamura, Ako Hosono, Hiroyuki Ogino, Gen Suzuki, Sunao Tokumaru, Takuya Sawada, Yoshiko Oshiro, Kazushi Maruo, Masashi Mizumoto

**Affiliations:** a Department of Radiation Oncology, Hyogo Ion Beam Medical Center Kobe Proton Center, Kobe, Hyogo, Japan; b Division of Radiation Oncology, Kobe University Graduate School of Medicine, Kobe, Hyogo, Japan; c Department of Radiation Oncology, University of Tsukuba, Tsukuba, Ibaraki, Japan; d Southern TOHOKU Proton Therapy Center, Southern TOHOKU Research Institute for Neuroscience, Koriyama, Fukushima, Japan; e Department of Pediatric Radiation Therapy Center, Hebei Yizhou Cancer Hospital, Bao Zhang Ji Di, Matou town, Zhuozhou, Hebei, PR China; f Fukui Prefectural Hospital Proton Therapy Center, Yotsui, Fukui city, Fukui, Japan; g Department of Radiation Oncology, National Cancer Center Hospital East, Kashiwa, Chiba, Japan.; h Department of Radiation Oncology, Nagoya Proton Therapy Center, Nagoya City University West Medical Center, Nagoya, Japan, Hirate-cho, Kita-ku, Nagoya, Japan; i Department of Radiology, Kyoto Prefectural University of Medicine, Kajii-cho, Kawaramachi-Hirokoji, Kamigyo-ku, Kyoto, Japan; j Department of Neurosurgery, Xuanwu Hospital Capital Medical University, Changchun St, Beijing, China; k Department of Medical Oncology, National Cancer Center Hospital East, Kashiwa, Chiba, Japan.; l Department of Radiation Oncology, Tsukuba Medical Center Hospital, Ibaraki, Japan; m Department of Biostatistics, Institute of Medicine, University of Tsukuba, Tsukuba, Ibaraki, Japan

**Keywords:** Paediatric intracranial ependymoma, Postoperative radiotherapy, Proton beam therapy, Treatment outcome, Prognostic factors, Secondary malignancies, Radiation necrosis, Retrospective study, Multi-centre study, East Asia

## Abstract

**Background:**

Postoperative proton beam therapy (PBT) has shown potential benefits for paediatric intracranial ependymoma; however, further evidence is needed. This study evaluated survival outcomes and toxicity in an East Asian multi-centre cohort.

**Materials and methods:**

We retrospectively analysed patients aged <20 years who underwent postoperative PBT for intracranial ependymoma at eight institutions in Japan and China between 2000 and 2023. Overall survival (OS) and progression-free survival (PFS) were estimated using the Kaplan–Meier method, while local failure was evaluated using the cumulative incidence function. Late adverse events were graded according to the Common Terminology Criteria for Adverse Events (CTCAE), version 4.0. Prognostic factors for OS, PFS, and local control (LC) were analysed.

**Results:**

Seventy-one patients were included (median age at PBT, 5.4 years). WHO Grade 3 ependymomas accounted for 73.2% of cases, and 59.2% were infratentorial. Gross total resection (GTR) was achieved in 64.8% of patients. The median prescribed dose was 59.4 Gy (RBE), and median follow-up was 45.5 months. The 3- and 5-year OS rates were 93.6% and 76.9%, respectively; PFS rates were 71.7% and 66.8%; and LC rates were 88.4% and 83.5%. Grade ≥ 2 late adverse events were uncommon; radiation necrosis in one patient (1.4%) and secondary malignancies in two (2.8%). On multivariate analysis, female sex and supratentorial location were independently associated with improved OS and PFS, and higher dose with improved OS. GTR tended to improve LC.

**Conclusions:**

Postoperative PBT is associated with favourable clinical outcomes and an acceptable toxicity profile in paediatric intracranial ependymoma.

## Highlights

• East Asian multi-centre study of postoperative PBT for paediatric ependymoma.
• Three- and five-year OS rates were 93.6% and 76.9%, respectively.
• Grade ≥ 2 late toxicities were uncommon, with low radiation necrosis incidence.
• Supratentorial location, female sex, and higher dose predict improved OS.
• GTR showed a non-significant trend toward improved local control.

## Introduction

Ependymal tumours are rare central nervous system tumours. According to the Central Brain Tumour Registry of the United States, the annual incidence of ependymal tumours is estimated at 0.43 patients per 100,000 population [1]. Intracranial ependymomas predominantly affect children younger than 15 years, with a slight male predominance. The median age at diagnosis is 5 years, with 25–40% of cases occurring before age 2. Intracranial ependymomas occur predominantly in the infratentorial region (in approximately two-thirds of cases), with the remainder in the supratentorial region [2], [3]. Tumour classification is based on histological and molecular assessment of tissue specimens according to the World Health Organization (WHO) 2021 classification [4]. Intracranial ependymomas exhibit heterogeneous clinical behaviour depending on tumour location, histology, and molecular subtype [5], [6]. In particular, the extent of resection has been reported to be one of the most important prognostic factors, with complete resection associated with favourable outcomes [5], [7], [8]. Despite advances in surgical techniques and radiotherapy (RT), local recurrence remains a major cause of treatment failure, highlighting the importance of achieving optimal local control (LC) during initial treatment [9]. Accordingly, current standard treatment for localised ependymoma consists of maximal tumour resection followed by RT, and European Association of Neuro-Oncology guidelines recommend postoperative local RT in paediatric patients with WHO grades II–III intracranial ependymomas regardless of residual tumour volume [5], [10], [11].

Traditionally, photon radiotherapy (XRT) has been used postoperatively for paediatric intracranial ependymoma. However, the proximity to critical brain structures may limit safe dose escalation, resulting in tumour underdosing [12]. In this context, a key advantage of proton beam therapy (PBT) over XRT is the Bragg peak, which enables highly conformal high-dose target irradiation while sparing adjacent normal tissues from low-to-intermediate radiation doses [13]. A dosimetric comparison between PBT and intensity-modulated radiotherapy (IMRT) showed that PBT significantly reduced doses to the cochlea, temporal lobes, whole brain, and hypothalamus, while maintaining similar target coverage [14]. With comparable tumour control, PBT may also provide better clinical preservation of hearing, endocrine, and cognitive function while reducing the risk of secondary malignancies and growth retardation [14], [15], [16], [17]. A meta-analysis demonstrated no significant difference in 5-year OS between XRT and PBT, whereas PBT was associated with a lower incidence of late adverse events [18]. Consequently, PBT has increasingly been adopted for paediatric brain tumours owing to its dosimetric advantage in sparing surrounding normal tissues.

However, the evidence regarding postoperative PBT for paediatric intracranial ependymomas remains limited. An important question remains whether PBT can reduce adverse events without compromising clinical outcomes. Furthermore, whether radiation-induced contrast enhancement or radiation necrosis is a clinically relevant concern in paediatric intracranial ependymomas remains an important issue requiring investigation. Recent studies suggest a higher incidence of temporary or clinically silent radiation-induced contrast-enhancing lesions compared with XRT, although multiple reports support the clinical utility of PBT [19], [20]. Therefore, we conducted an international multi-centre retrospective study in East Asia to evaluate the clinical outcomes and toxicity of postoperative PBT for paediatric intracranial ependymoma.

## Materials and methods

### Study design and patient eligibility

We conducted a retrospective, international, multi-centre study of paediatric patients with intracranial ependymomas treated with postoperative PBT. Eight PBT centres participated, seven in Japan and one in China. This multi-centre study was conducted in accordance with the Declaration of Helsinki and approved by the Institutional Review Board (approval number: R07–103) through a centralized review process. The requirement for written informed consent was waived due to the retrospective nature of the study, and an opt-out approach was used. Where applicable, institutional policies regarding consent for the use of clinical data for research purposes were followed. The inclusion criteria were: (i) treatment with PBT between January 2000 and December 2023; (ii) age < 20 years at the time of PBT; and (iii) histologically confirmed intracranial ependymoma. Exclusion criteria were: (i) prior irradiation to any site before the index PBT course; (ii) disease progression before postoperative PBT; and (iii) distant metastases or disseminated disease at diagnosis.

### Surgery and assessment of the extent of resection

Before postoperative PBT, patients were evaluated by a neurosurgeon to determine whether additional tumour resection was required according to the institutional treatment policy. Gross total resection (GTR) was defined as complete removal confirmed by surgical findings with no residual lesions on postoperative magnetic resonance imaging (MRI). Subtotal resection was defined as incomplete removal based on surgical findings, with postoperative MRI demonstrating a residual lesion less than 5 mm in thickness. Partial resection was defined as residual disease observed intraoperatively, with postoperative MRI showing a residual lesion measuring 5 mm or more in thickness [21].

### Treatment planning

PBT was delivered according to the treatment policy or protocol at each institution. Treatment planning for PBT used a computed tomography (CT)–based three-dimensional system. Each patient was immobilised in a custom-made thermoplastic mask in the supine position according to tumour location. Target volumes and organs at risk were delineated on fused CT–MRI images. The clinical target volume (CTV) included the gross tumour volume (GTV), the postoperative tumour bed, and any residual disease, with a 5–10 mm anatomically constrained margin. For passive-scattering PBT, a uniform planning target volume margin of 3–5 mm was applied in all directions. For active scanning PBT, the robust optimisation consisted of a 3–5 mm isotropic setup uncertainty and a 3.5% range uncertainty for the CTV. A constant relative biological effectiveness (RBE) of 1.1 was assumed for PBT. The prescribed dose was typically 59.4 Gy (RBE) in 1.8 Gy (RBE) fractions. Sedation was administered when clinically indicated.

### Follow-up and event ascertainment

Owing to the retrospective, international, multi-centre design, follow-up was conducted according to the routine clinical practice of each participating institution rather than a predefined, uniform protocol. Follow-up was performed according to each participating institution's standard protocol, which in general comprised clinical assessment and contrast-enhanced brain MRI every 3–4 months during the first 2–3 years after treatment, every 6 months thereafter until 5 years, and annually beyond 5 years, with spinal MRI performed when clinically indicated. Surveillance imaging obtained at referring institutions was forwarded to and reviewed at the treating PBT centre, and tumour recurrence and death were reported back to that centre. Tumour recurrence was diagnosed by the treating institution on the basis of serial imaging, with or without histological confirmation. Tumour and adverse events were retrospectively extracted from the existing medical records at each participating institution. Because surveillance was not standardised across institutions, the timing and modality of imaging and clinical assessment varied among patients, and evaluations requiring dedicated audiometric, endocrinological, or neuropsychological testing were performed at the discretion of the treating physician. As a substantial proportion of patients returned to their referring institutions after completion of PBT, the exact number of patients lost to follow-up could not be ascertained.

### Toxicity analysis

Because follow-up was not standardised, late adverse events were retrospectively assessed from available clinical and radiographic documentation. Late adverse events were assessed according to the Common Terminology Criteria for Adverse Events (CTCAE), version 4.0. For toxicity analysis, late adverse events of Grade ≥ 2 were collected. However, Grade 1 radiation necrosis was also included, as radiographic radiation necrosis, even when asymptomatic, remains clinically relevant after cranial irradiation. Radiation necrosis was assessed across the entire brain and diagnosed based on clinical and radiographic findings on follow-up imaging.

### Statistical analyses

Survival rates for overall survival (OS) and progression-free survival (PFS) were estimated using the Kaplan–Meier method. OS was defined as the time from the start of PBT to death from any cause. PFS was defined as the time from the start of PBT to tumour progression, recurrence, or death from any cause. Local failure was defined as local recurrence or local progression within the primary tumour bed or irradiated field. LC was defined as the absence of local failure, with time calculated from the start of PBT. For local failure, radiation necrosis (any-grade and Grade ≥ 2), and secondary malignancies, cumulative incidence functions were estimated, with death prior to these events treated as a competing risk. Univariate and multivariate Cox proportional hazards models were analysed for OS and PFS. For local failure, univariable and multivariable Fine–Gray sub-distribution hazard models were used. Covariates included age, sex, histological grade, tumour location, induction chemotherapy, number of surgeries, extent of resection, and prescribed dose. In this study, subtotal and partial resections were collectively categorised as “non-GTR” for comparison with GTR, because only five patients underwent partial resection, making separate detailed analyses statistically underpowered. In multivariate analyses, variables were selected using a stepwise procedure based on Wald test *p*-values (entry: *p* < 0.05; removal: *p* > 0.10). The significance level for all tests was *p* < 0.05, with 95% confidence intervals (CI). All analyses were conducted using R software, version 4.5.2 (R Core Team, Vienna, Austria) with relevant packages [22], [23], [24].

## Results

### Patient characteristics and treatment details

The patient characteristics are summarised in Table 1. A total of 71 patients were included in this study. The median age at PBT was 5.4 years (range, 0.5–17.9 years), and 36 patients (50.7%) were male. Histologically, 19 patients (26.8%) had WHO Grade 2 ependymoma, and 52 (73.2%) had Grade 3. The tumours were located in the supratentorial region in 29 patients (40.8%) and the infratentorial region in 42 (59.2%). Induction chemotherapy was administered to 19 patients (26.8%); none received concurrent chemoradiotherapy. The number of surgeries was one in 53 patients (74.6%) and two or more in 18 (25.4%); GTR was achieved in 46 patients (64.8%), and non-GTR was performed in 25 (35.2%). The median prescribed PBT dose was 59.4 Gy (RBE) (range, 50.4–63.0 Gy [RBE]).

**Table 1:** Patient characteristics and treatment details.

Characteristics			Number of patients (%) (*N* = 71)
Median age (range)			5.4 years (0.5–17.9)
Sex			
	Male		36 (50.7)
	Female		35 (49.3)
Performance status			
	0		31 (43.7)
	1		34 (47.9)
	2		5 (7.0)
	3		1 (1.4)
WHO histological grade			
	2		19 (26.8)
	3		52 (73.2)
Tumour location			
	Infratentorial		42 (59.2)
	Supratentorial		29 (40.8)
Induction chemotherapy			
	Present		19 (26.8)
	Absent		52 (73.2)
Number of surgeries			
	1		53 (74.6)
	≥2		18 (25.4)
Extent of resection			
	GTR		46 (64.8)
	Non-GTR		25 (35.2)
Median prescription dose (range)			59.4Gy (RBE) (50.4–63)

Abbreviations: GTR, gross total resection; RBE, relative biological effectiveness.

### Survival outcomes

**Fig. 1:**
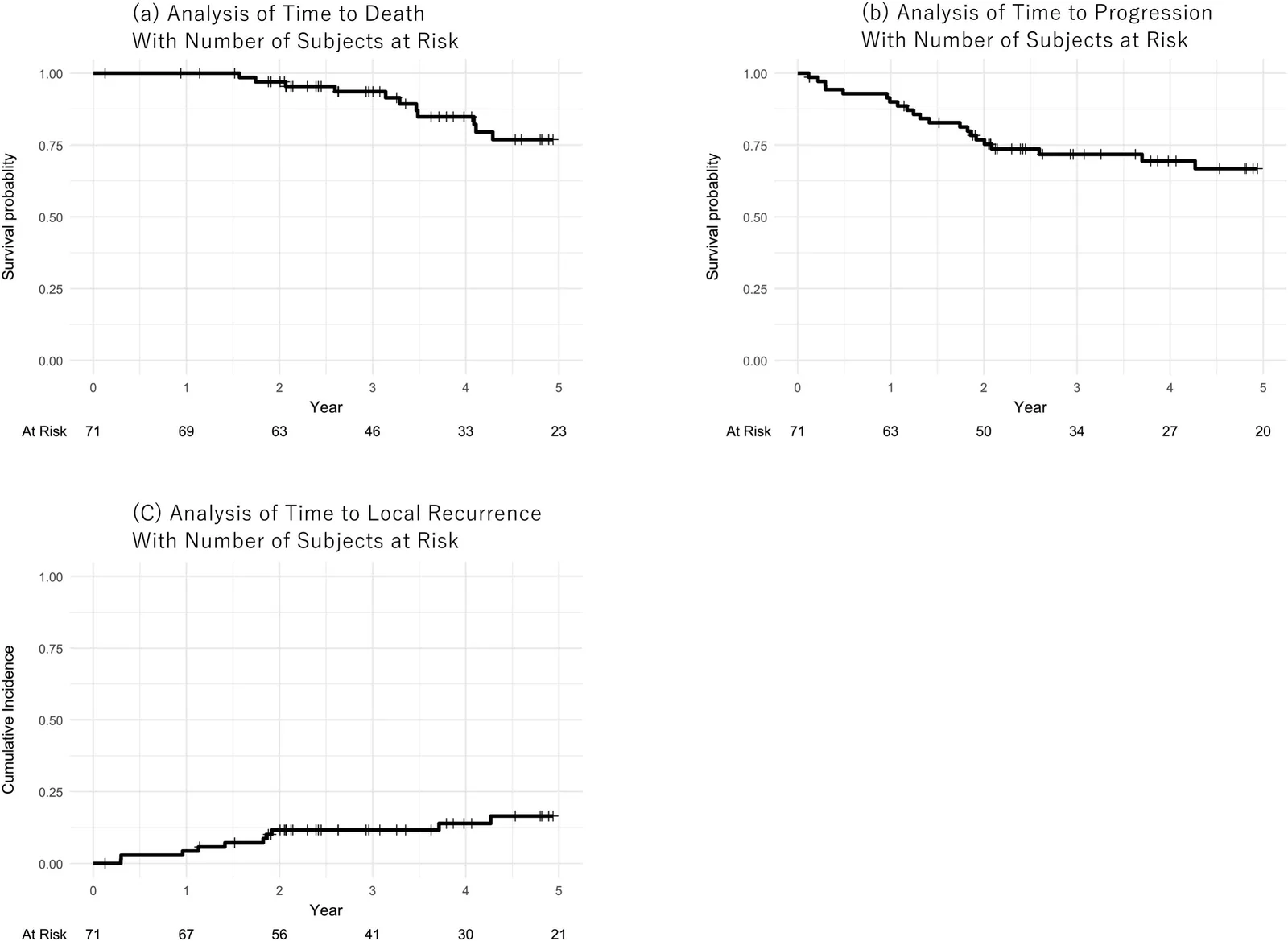
Rates of (a) overall survival, (b) progression-free survival, and (c) the cumulative incidence of local failure.

The median follow-up period was 45.5 months (range, 1.5–174.0 months). Event ascertainment relied on non-standardised institutional follow-up. The 3- and 5-year OS, PFS, and local failure rates were 93.6% (95% CI, 87.7%–99.9%) and 76.9% (95% CI, 65.4%–90.3%), 71.7% (95% CI, 61.7%–83.5%) and 66.8% (95% CI, 55.7%–80.0%), and 11.6% (95% CI, 5.4%–20.5%) and 16.5% (95% CI, 8.1%–27.4%), respectively (Fig. 1). The 3- and 5-year LC rates were 88.4% (95% CI, 79.5%–94.6%) and 83.5% (95% CI, 72.6%–91.9%). Disease progression occurred in 20 patients (28.2%): local failure only in 4 (5.6%), distant metastases only in 9 (12.7%), and both local and distant failure in 7 (9.9%). Overall, a local component of failure was present in 11 patients (15.5%) and a distant component in 16 (22.5%); the 7 patients with both failures are included in both of these figures.

### Prognostic factors

Univariate analysis identified female sex (*p* = 0.030), supratentorial tumour location (*p* = 0.037), GTR (*p* = 0.045), and a higher PBT dose (*p* = 0.043) as significantly positive prognostic factors for OS (Table 2). For PFS, female sex (*p* = 0.012), supratentorial tumour location (*p* = 0.024), and GTR (*p* = 0.016) were significantly positive prognostic factors. In contrast, no significant associations were observed between any prognostic factors and LC. On multivariate Cox regression analysis (Table 3), female sex (HR, 0.150; 95% CI, 0.038–0.586; *p* = 0.006), supratentorial tumour location (HR, 0.193; 95% CI, 0.050–0.745; *p* = 0.017), and higher PBT dose (HR, 0.857; 95% CI, 0.737–0.997; *p* = 0.045) were independently associated with better OS. In PFS, female sex (HR, 0.240; 95% CI, 0.091–0.635; *p* = 0.004) and supratentorial tumour location (HR, 0.174; 95% CI, 0.056–0.538; *p* = 0.002) were also independently associated with favourable outcome. GTR showed a trend toward improved LC; however, this was not statistically significant (HR, 3.179; 95% CI, 0.964–10.485; *p* = 0.058).

**Table 2:** Results of the univariable analysis.

Variable		N	5-year OS (%)	*P* value	5-year PFS (%)	P value	5-year LC (%)	P value
Age				0.644		0.748		0.390
Sex	Male	36	61.6	0.030	48.3	0.012	77.6	0.400
	Female	35	92.4		84.6		89.6	
WHO Histological grade	2	19	72.7	0.471	66.0	0.792	85.3	0.590
	3	52	78.8		67.7		83.0	
Tumour location	Infratentorial	42	66.6	0.037	54.8	0.024	76.4	0.089
	Supratentorial	29	89.5		81.1		91.7	
Induction chemotherapy	Present	19	69.1	0.505	73.7	0.933	89.5	0.760
	Absent	52	81.7		62.8		80.2	
Number of surgeries	1	53	83.3	0.085	68.8	0.711	81.3	0.490
	≥2	18	63.2		62.2		88.9	
Extent of resection	GTR	46	90.3	0.045	76.2	0.016	89.2	0.058
	non-GTR	25	51.0		49.9		73.9	
Prescription dose				0.043		0.054		0.600

Abbreviations: OS, overall survival; PFS, progression-free survival; LC, local control; GTR, gross total resection.

**Table 3:** Results of the multivariable analysis.

	OS			PFS			LC		
Variable	HR	95% CI	P value	HR	95% CI	P value	HR	95% CI	P value
Sex	0.150	0.038–0.586	0.006	0.240	0.091–0.635	0.004			
									
Tumour location	0.193	0.050–0.745	0.017	0.174	0.056–0.538	0.002			
									
Extent of resection							3.179	0.964–10.485	0.058
									
Prescription dose	0.857	0.737–0.997	0.045						

Abbreviations: OS, overall survival; PFS, progression-free survival; LC, local control; HR, hazard ratio; CI, confidence interval.

### Treatment-related toxicity

**Fig. 2:**
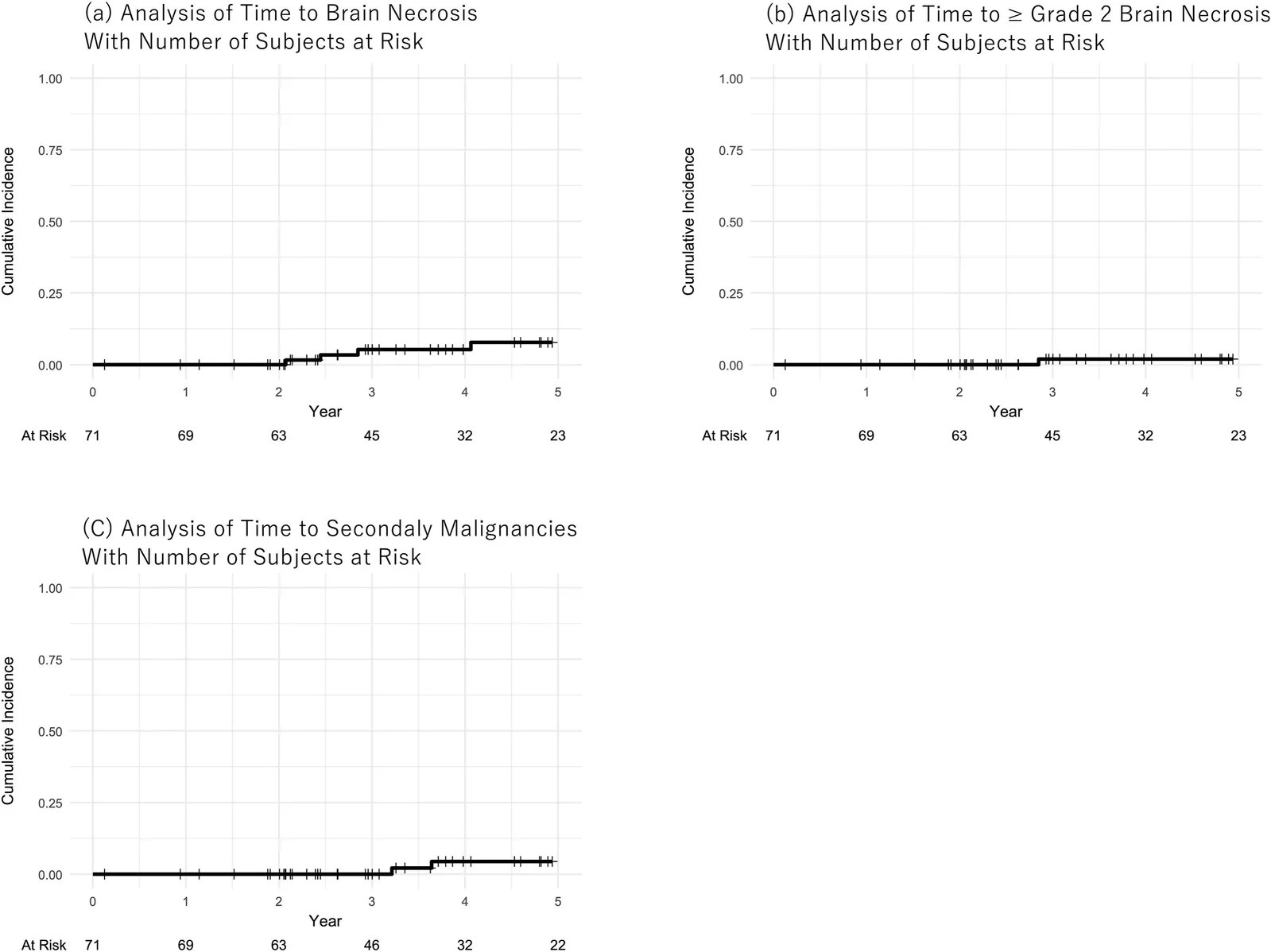
Cumulative incidence functions of (a) any-grade radiation necrosis, (b) Grade ≥ 2 radiation necrosis, and (c) secondary malignancies.

Across all cases, Grade 2 late adverse events were observed in four patients, including alopecia in two patients (2.8%), hearing impairment in one patient (1.4%), and radiation necrosis in one patient (1.4%). No Grade ≥ 3 late adverse events were observed. One patient with Grade 2 hearing impairment did not receive induction chemotherapy. Radiation necrosis of any grade was observed in seven patients. Grade 1 radiation necrosis occurred in six patients (8.5%) between 0.5 and 3.8 years after the index PBT, whereas Grade 2 radiation necrosis occurred in one patient at 2.8 years after the index PBT (Fig. 2). The patient with Grade 2 radiation necrosis had undergone subsequent salvage re-irradiation during follow-up for local recurrence; accordingly, this Grade 2 event cannot be attributed to the index PBT alone. Among the Grade 1 cases, one (17%) similarly occurred in a patient who had received salvage re-irradiation, whereas the remaining five (83%) occurred after the index PBT alone. Secondary malignancies were observed in two patients (2.8%), at 3.2 and 3.6 years after PBT, respectively (Fig. 2). One patient developed glioblastoma within the irradiation field, confirmed pathologically after surgical resection. Another patient developed a brainstem lesion without histological confirmation; it formed a well-defined mass and was clinically diagnosed as a secondary malignancy.

## Discussion

To the best of our knowledge, this is the first East Asian international multi-centre collaborative study evaluating clinical outcomes and toxicity of postoperative PBT for paediatric intracranial ependymomas.

In the present study, postoperative PBT demonstrates favourable outcomes, with 3-year OS, PFS, and LC rates of 93.6%, 71.7%, and 88.4%, respectively, and 5-year rates of 76.9%, 66.8%, and 83.5%, respectively. These results are comparable to previous studies on postoperative PBT for paediatric intracranial ependymomas [8], [17], [25], [26], [27], [28] (Table 4). In a direct comparison of XRT and PBT in paediatric intracranial ependymoma, Mizumoto et al. reported equivalent outcomes, with 3-year OS/LC rates of 81.2%/77.3% for XRT and 91.1%/82.6% for PBT, and 5-year rates of 73.8%/72.6% for XRT and 84.7%/79.0% for PBT, respectively [17].

**Table 4:** Recent literature review of postoperative PBT in paediatric intracranial ependymomas.

Reference	N	Median f/u period (month)	PBT Median dose (Gy [RBE])	OS	PFS	LC	Toxicity (%)
Ares et al. (2016, Switzerland) [25]	50	43	59.4	84.0% (5-year)	N/A	78.0% (5-year)	Brainstem necrosis (Gr5): 2% Hearing impairment (Gr4): 4% Hormone deficiency (Gr ≥ 2): 6% Hypothyroidism (Gr ≥ 2): 6%
Indelicato et al. (2018, United States) [26]	179	38	59.4	90.4% (3-year)	75.9% (3-year)	85.4% (3-year)	Radiation necrosis (Gr ≥ 2): 5.5% Hormone deficiency (Gr ≥ 2): 7.3% Hearing impairment (Gr ≥ 3): 6.1%
Indelicato et al. (2021, United States) [8]	386	60	55.8	84.7% (5-year)	68.4% (5-year)	79.2% (5-year)	Radiation necrosis (Gr ≥ 2): 4% Secondary malignancies: 1.3% Hearing impairment (Gr ≥ 3): 5.4%
Peters et al. (2022, Germany) [27]	105	22	59.4	93.7% (3-year)	55.6% (3-year)	74.1% (3-year)	Hearing impairment (Gr ≥ 2): 3.8% Other late toxicity (Gr ≥ 2): 6.7%
Mizumoto et al. (2024, Meta-analysis) [17]	831	N/A	N/A	84.7% (5-year)	N/A	79.0% (5-year)	N/A
Le Reun et al. (2025, Switzerland) [28]	119	63	59.4	82.2% (5-year)	63.5% (5-year)	70.4% (5-year)	Radiation necrosis (Gr ≥ 2): 1.6% Secondary malignancies: 3.4% Hormone deficiency (Gr ≥ 2): 12.6% Hearing impairment (Gr ≥ 3): 4.2% Other late toxicity (Gr ≥ 2): 5%
Present study (2026, Japan/China)	71	45	59.4	93.6% (3-year) 76.9% (5-year)	71.7% (3-year) 66.8% (5-year)	88.4% (3-year) 83.5% (5-year)	Radiation necrosis (Gr ≥ 2): 1.4% Secondary malignancies: 2.8% Hearing impairment (Gr ≥ 2): 1.4% Alopecia (Gr ≥ 2): 2.8%

Abbreviations: OS, overall survival; PFS, progression-free survival; LC, local control; PBT, proton beam therapy; f/u, follow-up; RBE, relative biological effectiveness; Gr, Grade; N/A, Not applicable.

In multivariate analysis, supratentorial location, higher PBT dose, and female sex were favourable prognostic factors for OS, while supratentorial location and female sex were associated with improved PFS. GTR showed a non-significant trend toward improved LC. Previous studies show that extent of resection is a key prognostic factor in paediatric intracranial ependymoma, with better outcomes after GTR. MacDonald et al. and Indelicato et al. reported that subtotal resection was associated with inferior LC, and Indelicato et al. also demonstrated worse PFS and OS [8], [14]. In the present study, however, we did not observe a statistically significant difference in LC between the GTR and non-GTR groups. A possible explanation is the relatively lower GTR rate in our cohort (64.8% vs. 85% and 76% in previous studies) and the relatively small sample size, which may have limited statistical power. In contrast, Indelicato et al. reported significant differences in OS and PFS between the two groups, possibly reflecting their larger sample size [8]. Infratentorial lesions were associated with a poorer prognosis, likely reflecting both the anatomical and molecular characteristics of the disease. Indelicato et al. reported that posterior fossa tumours were associated with inferior PFS and OS, consistent with our findings [8]. Fourth ventricular tumours often involve critical brainstem structures, limiting safe GTR, and posterior fossa ependymomas frequently belong to the unfavourable Posterior fossa ependymoma group A (PF-EPN-A) molecular subtype [6], [29]. Consistent with these findings, the GTR rate in our cohort was lower for infratentorial than supratentorial tumours (60% vs. 72%). The lack of molecular subgroup information in the present study may also have influenced the interpretation of these results. Although higher radiation doses were associated with improved OS in our cohort, previous studies have suggested a limited dose-dependency, particularly following GTR. Patteson et al. reported no dose–response relationship, with similar 7-year LC between ≤54 Gy (RBE) and > 54 Gy (RBE) [30], and a meta-analysis by Rose et al. likewise found no differences in LC, event-free survival, or OS by dose [31]. This discrepancy may partly reflect the slightly higher proportion of non-GTR cases in our cohort, although the exact cause remains unclear. Male sex has been associated with inferior PFS and OS, as reported by Indelicato et al., possibly reflecting a higher prevalence of the unfavourable PF-EPN-A molecular subtype in males [8]. The impact of sex on outcomes remains inconclusive and warrants further molecular investigation.

Regarding adverse events, Grade ≥ 2 late toxicities in our cohort were infrequent, including alopecia (2.8%), hearing impairment (1.4%), radiation necrosis (1.4%), and secondary malignancies (2.8%). These results were lower than or consistent with those reported in previous postoperative PBT series for paediatric intracranial ependymoma [8], [25], [26], [27], [28] (Table 4). In a meta-analysis of paediatric brain tumour patients, Kiss-Miki et al. also reported reduced late toxicities, including endocrine and neurocognitive effects, with PBT compared with those seen with XRT [18]. PBT may reduce radiation-induced secondary malignancies due to a reduced integral dose to normal tissues; a matched cohort study showed a lower incidence with PBT than XRT (5.2% vs 7.5%; adjusted HR 0.52) [32]. This potential advantage is particularly important in paediatric patients with long life expectancy. In contrast, Indelicato et al. reported Grade ≥ 2 radiation necrosis following PBT in 4%, which is slightly higher than the 2.5% incidence of treatment-related brainstem necrosis reported in XRT-based series by Merchant et al. [8], [21]. These findings suggest that the incidence of radiation necrosis after PBT may be relatively higher than that observed in XRT-based series; however, direct comparisons should be interpreted with caution due to differences in study populations and treatment techniques. Radiation necrosis following PBT is further discussed in the next section.

Radiation necrosis is one of the most clinically significant adverse events following cranial PBT. Kralik et al. reported imaging findings indicative of radiation necrosis in 31% of paediatric brain tumours treated with PBT, with symptomatic necrosis in 7.7%, indicating that most radiographic changes remain asymptomatic [33]. Gunther et al. reported no significant difference in symptomatic radiation necrosis between XRT and PBT (8.5% vs. 11%); however, MRI changes were more frequent and occurred earlier with PBT than with IMRT (43% vs. 17%; median onset, 3.8 vs. 5.3 months) [20], [34]. A possible explanation for more frequent MRI changes after PBT is the distal-edge effect, whereby high-RBE protons extend into adjacent normal tissues such as the brainstem; however, despite this theoretical concern, clinically the incidence of Grade ≥ 2 brainstem necrosis remains low [35]. A recent systematic review and meta-analysis reported symptomatic brainstem toxicity after PBT in paediatric brain tumours in 0.7% of cases, with a rare brainstem-related mortality rate (0.5%), thereby supporting the overall safety of PBT [36]. However, the risk appears to vary by tumour histology, with ependymomas showing a higher risk of brainstem toxicity than tumours such as medulloblastoma, possibly due to higher focal doses to the posterior fossa; Upadhyay et al. reported a 5.8% incidence after postoperative PBT in paediatric intracranial ependymomas [37]. Table 4 summarises the reported incidence of Grade ≥ 2 radiation necrosis following postoperative PBT in paediatric patients with intracranial ependymomas [8], [25], [26], [28]. The incidence of Grade ≥ 2 radiation necrosis in our cohort was low and comparable with that reported in previous series. Taken together, these East Asian real-world data demonstrate a relatively low incidence of radiation necrosis following postoperative PBT in paediatric intracranial ependymoma, further reinforcing the favourable safety profile of PBT in this population.

This study has four main limitations. First, the retrospective nature of the study may have introduced selection bias, limited control over confounding variables, and reduced the precision of treatment effects and adverse events. Second, the lack of molecular subtype classification prevents risk stratification based on contemporary molecular profiling, which has been shown to have important prognostic implications in paediatric ependymoma. However, although molecular classification was unavailable, our cohort reflects real-world clinical practice during the study period. Third, as a multi-centre study, the heterogeneity in radiation equipment and treatment planning across institutions may have affected treatment delivery, toxicity evaluation, and disease control. In particular, the prescription dose was not standardised and varied across institutions and treatment eras (50.4–63 Gy(RBE)). Doses above 59.4 Gy(RBE) were prescribed mainly in earlier cases, before 59.4 Gy(RBE) became the widely accepted standard prescription dose [21], and were determined at the discretion of each institution. This lack of a uniform dose criterion should be taken into account when interpreting our clinical outcomes. Relatedly, standardised dose–volume histogram (DVH) data were not uniformly collected across institutions; consequently, a site-specific, dose–volume analysis of radiation necrosis—including correlation with brainstem dose parameters such as Dmax, Dmean, and V54/V55—could not be performed. Notably, the observed necrotic events did not necessarily arise within the brainstem, and characterising them specifically as brainstem necrosis would not accurately reflect the events observed. Fourth, follow-up was not governed by a common protocol and was performed according to the routine clinical practice of each participating institution; consequently, the interval between surveillance examinations varied among patients, and the exact number of patients lost to follow-up could not be ascertained. Because recurrence was detected at institution-dependent imaging intervals, the timing of recurrence may have been ascertained with differing precision across centres, although this is unlikely to have materially affected the estimation of long-term outcomes. Surveillance imaging performed at referring institutions was routinely forwarded to the treating proton centres, and survival, recurrence, radiation necrosis, and intracranial second tumours are therefore considered to have been adequately captured; consistent with this, the incidence of major late toxicities, including brainstem injury and secondary malignancies, was comparable to that reported in previous proton therapy series. By contrast, toxicities requiring dedicated clinical or laboratory evaluation—including alopecia, hearing impairment, endocrine dysfunction, and neurocognitive outcomes—were assessed at the discretion of the treating physician rather than by protocol, and their reported incidence should be regarded as a lower bound rather than a precise estimate. In addition, the median follow-up of 45.5 months is relatively short given the propensity of intracranial ependymoma for late recurrence, and the 5-year outcome estimates should therefore be regarded as preliminary. Although 23 of 71 patients (32%) remained at risk at 60 months—exceeding the commonly applied threshold below which Kaplan–Meier estimates become unstable—continued follow-up is warranted to reliably characterise late recurrence and survival. The variability in dose selection and follow-up strategies observed across participating institutions highlights the need for standardised treatment and surveillance protocols in future collaborative studies. Nevertheless, despite these limitations, the current study remains highly valuable, as it is the first East Asian international collaborative analysis to suggest the potential utility of postoperative PBT for paediatric intracranial ependymoma. These results contribute to the growing body of evidence supporting the role of postoperative PBT in this population. Future studies with larger cohorts and longer follow-up are warranted to validate these findings.

## Conclusions

In this East Asian multi-centre retrospective study, postoperative PBT for paediatric intracranial ependymomas is associated with favourable outcomes and an acceptable toxicity profile, with low incidences of radiation necrosis and secondary malignancies. Prospective studies with longer follow-up periods are required to validate these findings.

## Declaration of generative AI and AI-assisted technologies in the manuscript preparation process

The authors declare that no generative AI or AI-assisted technologies were used in the preparation of this manuscript.

## Funding

This work was supported by JSPS KAKENHI Grant Number JP22K07764.

## CRediT authorship contribution statement

**Akifumi Kajihara:** Writing – review & editing, Writing – original draft, Visualization, Methodology, Investigation, Data curation, Conceptualization. **Yusuke Demizu:** Writing – review & editing, Supervision, Methodology, Conceptualization. **Takashi Saito:** Writing – review & editing, Investigation. **Yusuke Azami:** Writing – review & editing, Investigation. **Shosei Shimizu:** Writing – review & editing, Investigation. **Sae Matsumoto:** Writing – review & editing, Investigation. **Yuichi Hiroshima:** Writing – review & editing, Investigation. **Hiromitsu Iwata:** Writing – review & editing, Investigation. **Norihiro Aibe:** Writing – review & editing, Investigation. **Hideyuki Sakurai:** Writing – review & editing, Funding acquisition. **Masao Murakami:** Writing – review & editing, Investigation. **Gao Zeng:** Writing – review & editing, Investigation. **Hiroyasu Tamamura:** Writing – review & editing, Investigation. **Ako Hosono:** Writing – review & editing, Investigation. **Hiroyuki Ogino:** Writing – review & editing, Investigation. **Gen Suzuki:** Writing – review & editing, Investigation. **Sunao Tokumaru:** Writing – review & editing, Investigation. **Takuya Sawada:** Writing – review & editing, Validation, Data curation. **Yoshiko Oshiro:** Writing – review & editing, Validation, Data curation. **Kazushi Maruo:** Writing – review & editing, Supervision, Methodology, Formal analysis, Conceptualization. **Masashi Mizumoto:** Writing – review & editing, Supervision, Project administration, Methodology, Conceptualization.

## Ethical statement

Study approval statement: This multi-centre study was approved by the Institutional Review Board through a centralized review process (approval number: R07–103).

Consent for publication: The requirement for written informed consent was waived due to the retrospective nature of the study, and an opt-out approach was used. Where applicable, institutional policies regarding consent for the use of clinical data for research purposes were followed.

## Declaration of competing interest

The authors declare that they have no known competing financial interests or personal relationships that could have appeared to influence the work reported in this paper.
